# A meta-analysis of cases of Rosai Dorfman disease reported on the African continent and a description of two cases from a tertiary academic hospital in Johannesburg, South Africa

**DOI:** 10.11604/pamj.2023.45.130.40709

**Published:** 2023-07-18

**Authors:** Garrick Edouard Laudin, Atul Baldev Lakha, Nishal Dullabh, Reena Mohanlal, Romana Jassat, Muhammed Faadil Waja, Vinitha Philip

**Affiliations:** 1 Department of Medicine Clinical Haematology Unit, Chris Hani Baragwanath Academic Hospital and Faculty of Health Sciences, University of the Witwatersrand, Johannesburg, South Africa,; 2 Department of Medicine, Charlotte Maxeke Academic Hospital and Faculty of Health Sciences, University of the Witwatersrand, Johannesburg, South Africa,; 3 Anatomical Pathology, School of Pathology, University of the Witwatersrand, National Health Laboratory Service, Johannesburg, South Africa

**Keywords:** Lymphadenopathy, histiocytic disorders, sinus histiocytosis with massive lymphadenopathy (SHML), Rosai-Dorfman-Destombes disease (RDD)

## Abstract

Rosai-Dorfman-Destombes disease (RDD) is a rare non-Langerhans cell histiocytosis characterized by the accumulation of activated histiocytes within affected tissues. The original haematopathological description of RDD has links to the late South African born haematopathologist, Ronald Dorfman, with a descriptive account of two cases of the disease treated at Chris Hani Baragwanath Academic Hospital are described herein. Alongside the two case descriptions is a meta-analysis of 149 published cases from the African continent. Sequential literature searches were performed on Google Scholar and PubMed with the search terms “sinus histiocytosis with massive lymphadenopathy”, “Rosai-Dorfman disease”, “Rosai-Dorfman Destombes” and “lymphadenopathy” together with the name of each individual country on the African continent, from Algeria to Zimbabwe. All possible cases of RDD reported in published literature from Africa were captured on a Microsoft Excel spreadsheet recording details, where available, of demographics, nodal (nodal groups) or extra-nodal disease as well as treatment. Of the 54 African countries on the continent, published data was available from half of these countries (n=27). Nigeria (35), Tunisia (25) and South Africa (23) contributed the majority of cases for data collection with a clear paucity of reportable information available from Central Africa. Of the 149 cases from the African continent, the majority were from patients aged ten years and younger with a decrease in reported cases in patients with increasing age. The mean age at diagnosis was 25.66 years [95% CI: 21.81-29.51] with a median age of diagnosis of 24.5 years. The youngest patient in the series was 3 months old and the oldest patient aged 72 (range 71.75 years, IQR 31). The cases reported were fairly split between males and females with a male-to-female ratio of 1.07: 1. HIV seropositivity was reported in seven patients (4.8%) and no HIV results were available in 104 patients (71.2%). Disease presentation was split between nodal disease in 43% of patients (n=64), Extra nodal (EN) disease in 32.9% (n=32), mixed (nodal/EN) disease in 11.4% (n=17) and unknown in 12.8% (n=19). Fever was present in 18.1% (n=27) of cases. Hepatic enlargement was noted in nine patients (6%) and splenic enlargement in four patients (2.7%). Commonly ascribed sites of EN disease, in descending order, were skin and soft tissue, ocular, ear/nose/throat (ENT), abdominal organ(s), bone, lung/pleura, brain parenchyma (including dura), endocrine glands, spine, breast, pericardium, pseudotumour formation (unspecified site), joint(s), peripheral nerves and genitourinary tract disease. The upfront administration of glucocorticosteroids was seen in the majority of cases. Rosai-Dorfman-Destombes, although a rare disorder, should be considered as a differential diagnosis in patients with massive bilateral cervical lymphadenopathy and is confirmed with accompanying pathological changes on microscopic and immunohistochemical examination of biopsy specimens. The role of infection, particularly HIV infection, is considered to be a possible contributor to the pathogenesis of RDD and HIV testing in patients from areas of high HIV endemicity with co-existing RDD should be undertaken. Consideration for mycobacterium tuberculosis infection in patients with generalized significant lymphadenopathy still remains an important differential for massive lymphadenopathy and requires confirmation by appropriate microbiological investigations. The treatment landscape in RDD is limited in many resource-poor settings, with the upfront use of glucocorticosteroids employed routinely in the majority of cases.

## Introduction

Rosai-Dorfman-Destombes disease (RDD) is a rare non-Langerhans cell histiocytosis, characterized by the accumulation of activated histiocytes within affected tissues [1,2]. The haematopathological description of RDD has links to the South African trained haematopathologist, the late Ronald Dorfman. Dorfman, born in Johannesburg (South Africa), was an alumnus of the University of the Witwatersrand (“Wits”) having graduated in 1948 with his undergraduate medical degree. Dorfman later pursued his post-graduates studies in haematopathology at the South African Institute for Medical Research (SAIMR) and served as a pathologist and a lecturer at the University of the Witwatersrand from 1959 to 1962 [2]. In 1963, Dorfman relocated to the United States of America after an appointment to Washington University in St. Louis, Missouri [2]. It was here that Dorfman and Dr Juan Rosai shared identical examples of a rare, benign lymph node disorder, eponymously referred to as Rosai-Dorfman disease, or by the descriptive term sinus histiocytosis with massive lymphadenopathy (SHML) [3]. The inclusion of the name Destombes to the disease, is after the original description in 1965 of the lymph node pathology in four children with the disease, by a French pathologist, Pierre Paul Louis Lucien Destombes [4]. Destombes initially described the disease as “adenitis with lipid excess” after noting the lipid content of histiocytes on histological examination of lymph node tissue 24. A description of two cases of RDD treated in the department of Clinical Haematology at Chris Hani Baragwanath Academic Hospital (Johannesburg, South Africa) are described below. A literature review and analysis of case reports of the disease described on the African continent is also described herein and serves to create awareness of other diagnostic considerations in massive lymphadenopathy.

## Methods

Sequential literature searches were performed on Google Scholar and PubMed with the search terms “sinus histiocytosis with massive lymphadenopathy”, “Rosai-Dorfman disease”, “Rosai-Dorfman Destombes” and “lymphadenopathy” together with the name of each individual country on the African continent, from Algeria to Zimbabwe. All possible cases of RDD reported in published literature from Africa were captured on a Microsoft Excel spreadsheet recording details, where available, of demographics, nodal or extra-nodal disease as well as treatment. Details from English and non-English articles were included in the data-set and articles from Francophone African countries were translated into English using Google translate. The references for the cases utilized in the meta-analysis are available from the author on request.

### Description of two cases at Chris Hani Baragwanath academic hospital

**Figure 1:**
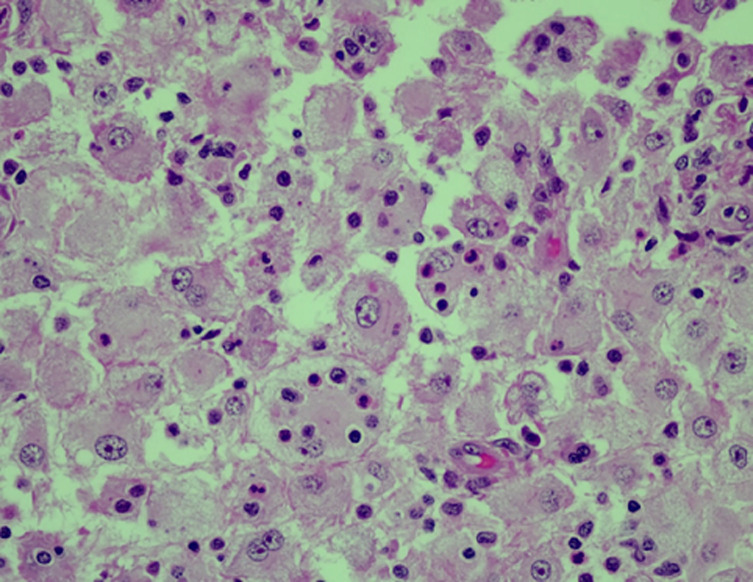
high power image showing infiltrate of histiocytes with emperipolesis on tissue biopsy from patient 1 (Hematoxylin and eosin stain)

**Case 1:** patient one is a 36-year-old female, Human Immunodeficiency virus (HIV) positive and virally suppressed on combined antiretroviral therapy (cART). Pertinent prior medical history includes a history of a left leg deep vein thrombosis (DVT). She reported a six-month history of a right-sided neck mass (lymph node conglomerate), with subsequent development of a nasal-mass accompanied by obstructive symptoms. There were no accompanying constitutional symptoms. Histological examination of the lymph node tissue (Figure 1) noted a characteristic histiocytic infiltrate with emperipolesis. An incisional biopsy of a cervical lymph node with accompanying nasal mass biopsy, were notable for sinus histiocytosis with massive lymphadenopathy (SHML) or RDD. Her initial blood work, including a full blood count (FBC), was unremarkable with no histiocytic infiltrate reported on bone marrow biopsy specimens. Initial treatment with methotrexate (MTX) (20 mg/week), mercaptopurine (MCP) (50 mg/day) and prednisone (1mg/kg) was commenced with regular monitoring of lymph node size undertaken at our Clinical Haematology outpatient clinic. The patient continues her follow up at our department at the time of publication.

**Case 2:** patient two was referred to the Clinical Haematology Department at Chris Hani Baragwanath Academic Department (Soweto, Johannesburg) with a history of generalized lymphadenopathy with prominent right-sided neck adenopathy. Histological confirmation of RDD on excised nodal tissue by immunophenotyping (S100 positive, CD68 positive) prompted the initiation of intravenous and subsequent oral glucocorticosteroids (GCS) at her referral hospital, prior to transfer to our center. Bone marrow examination was negative for a histiocytic infiltrate. Extra-nodal (EN) disease in the form of liver lesions and vertebral (thoracolumbar) lytic lesions (T7, T8, T11, L4, L5), in addition to nodal disease, was present on computerized tomography (CT) imaging. After 12 cycles of combination chemotherapy bilateral cervical chain lymphadenopathy of the right infra-clavicular region (10 x 9.5 mm) and left supraclavicular (1.6 x 14.4 mm) region was noted on follow-up imaging. Disease relapse with the development of additional lymphadenopathy (left supraclavicular) and a nasal mass, histologically confirmed RDD, whilst on MTX and MCP therapy, prompted the later addition of thalidomide to her current treatment regimen.

**Ethical approval:** all procedures performed in studies involving human participants were in accordance with the ethical standards of the institutional and/or national research committee and with the 1964 Helsinki declaration and its later amendments or comparable ethical standards. Ethics clearance has been obtained from the Human Research Ethics Committee (HREC) of the University of the Witwatersrand, Johannesburg (South Africa) (Ref: M230450).

## Results

Of the 54 African countries on the continent, published data was available from half of these countries (n=27). Nigeria (35), Tunisia (25) and South Africa (23) contributed to the majority of cases for data collection with a clear paucity of reportable information available from Central Africa. From the time period of reported cases between 1974 and 2015 it is notable that there has steadily been an increase in the number of reported cases over time with an exponential growth of publications in the latter years. Of the 149 cases from the African continent, the majority were from patients aged ten years and younger, with fewer cases reported in older individuals. The mean age at diagnosis was 25.66 years [95% CI: 21.81 - 29.51] with a median age of diagnosis of 24.5 years. The youngest patient in the series was 3 months old and the oldest patient was aged 72 (range 71.75 years, IQR 31). The cases reported were fairly split between males and females with a male-to-female ratio of 1.07: 1. HIV seropositivity was reported in seven patients (4.8%) and no HIV results were available in 104 patients (71.2%). Disease presentation was split between nodal disease in 43% of patients (n=64), extra-nodal (EN) disease in 32.9% (n=32), mixed (nodal/EN) disease in 11.4% (n=17) and unknown site in 12.8% (n = 19). Fever was present in 18.1% (n=27) of cases. Hepatic enlargement was noted in nine patients (6%) and splenic enlargement in four patients (2.7%). Commonly ascribed sites of EN disease in our review, in descending order, were skin and soft tissue (37.04%), ocular (13.58%), ear/nose/throat (ENT) (9.87%), abdominal organs and serosa (8.64%), bone (6.17%), lung/pleura (4.94%), brain parenchyma and dura (3.70%), endocrine glands (3.70%), spine (1.37%), breast (1.37%), pericardium (1.37%), pseudotumour formation (unspecified site) (0.68%), joint (0.68%), peripheral nerves (0.68%) and genitourinary tract disease (0.68%). Treatment modalities employed in treatment included upfront administration of glucocorticosteroids in the majority of cases (32.9%), surgical excision (14.1%), anti-tuberculous therapy (11.4%), chemo-immunotherapy (8.7%), a “watch-and-wait” approach (2%), immunosuppressants (1.3%), radiation therapy (1.3%) and combination anti-retroviral therapy (1.3%).

## Discussion

Rosai-Dorfman-Destombes is a rare non-Langerhans cell histiocytosis characterized by accumulation of activated histiocytes within affected tissues [4]. Rosai-Dorfman-Destombes affects both children and young adults with an average age of onset of 20.6 years and occurs more commonly in African patients with a slight male predominance [1,5]. The classic presentation of RDD however is in children less than ten years of age, which largely mirrored the findings in our meta-analysis [5]. Literature from one patient cohort of 64 patients noted a female preponderance of the disease with a male-to-female ratio (M: F) of 1: 1.5 [3]. Despite the reported frequency being greater in males, our analysis of cases reported on the African continent revealed a relatively equal incidence in males and females with a M: F ratio of 1,07: 1. The classical presentation of the disease is massive lymphadenopathy, typically painless, located within the cervical region [4,5]. Extra-nodal disease is noted in over 40% of patients which mirrors data from Goyal *et al*. and their study of 64 patients in which 67% of patients had EN disease [3,4]. Although regarded as rare, close to one-third of patients in our meta-analysis had EN disease at presentation [5]. Extra-nodal disease in our analysis of 149 cases largely paralleled that reported in mainstream literature, with common sites including the skin, nasal cavity, bone, orbital tissue and central nervous system (CNS) (including the dura) [1,5]. The diagnosis of RDD whilst not finite, is a composite of: (1) histological appearance of extensive sinusoidal expansion with sinusoids filled with numerous large histiocytes [1,4] and (2) the presence of histiocytes positive for S100, CD68 and CD163 and an absence of the cell markers seen in Erdheim-Chester disease (ECD), Juvenile xanthogranuloma (JXG) or Langerhans cell histiocytosis (LCH) [5]. Emperipolesis, or the trafficking of lymphocytes or plasma cells within the cytoplasm of RDD histiocytes, is a histological finding on sectioned tissue that may support a diagnosis of RDD and is appreciated in Figure 1 below [5]. The incited aetiologies of RDD are heterogenous and the disease frequently occurs in the setting of autoimmune disease, hereditary syndromes and malignant disease [4]. The role of infection, particularly HIV infection, is considered to be a possible contributor to the pathogenesis of RDD therefore HIV testing in patients from areas of high HIV endemicity with co-existing RDD should be undertaken [4,6]. Global data from the United Nations Programme on HIV/AIDS (UNAIDS) noted Eastern and Southern Africa as major contributors to the high number of new HIV infections in 2021 [7].

Notable from the analyses of cases from Africa was a lack of serological testing for HIV, with only seven cases confirmed HIV seropositive. Other viruses associated with RDD include herpes viruses, Epstein-Barr virus and cytomegalovirus [4]. The pathogenesis of RDD is associated with activating mutations in the mitogen-activated protein kinase extracellular signal-regulated kinase (MAP-ERK) pathway [1,3,5]. Germline mutations in solute carrier family 29 member 3 (SCL29A3) have been described in hereditary syndromes including H-syndrome and Faisalabad syndrome with the two diseases displaying findings compatible with RDD [8]. A single case report from Tunisia alluded to the marked phenotypic variability in a family of five members who each harboured the SCL29A3 gene mutation, with the family members presenting with overlapping features of H-syndrome and familial RDD [9]. There is no standardized treatment regimen that is uniformly adopted when managing RDD and the optimal duration of glucocorticosteroids (GCS) or other systemic therapies for RDD has not yet been established. Patients with the classic manifestation of RDD typically present with localized lymphadenopathy or subcutaneous nodules, with slow disease progression patterned with remissions and relapses occurring over many years [5]. The use of GCS upfront in therapy was noted in close to a third of the case reports from metanalysis. The utility of steroids in reducing the size of nodal masses and improving symptomatology is offset by the deleterious effects of high doses of steroids, disease relapse on steroid interruption, as well as lack of a durable response in extra-nodal disease [4,5]. The patient in case two above represents a subgroup of patients in whom the clinical course is largely unpredictable and protracted. This patient received multiple courses of chemotherapy administered over a period spanning six years and remains clinically stable on MTX, MCP and thalidomide [4]. With the discovery of mitogen activated protein kinase extracellular signal-regulated kinase (MAP-ERK) mutations in RDD as well as other histiocytic neoplasms, novel targeted therapy with MEK-inhibitors such as cobimetinib has been utilised in cases of relapsed or refractory disease, or in cases of RDD with central nervous system (CNS) involvement [5]. Whilst the general diagnostic approaches to lymphadenopathy vary and were not always alluded to in each case of RDD in the literature, fine needle aspirate (FNA) specimens with cytological analysis are largely regarded as diagnostic triage tools. Fine needle aspirate has limited utility overall, owing to retrieval of a small sample size and lack of detail on nodal architecture. A negative result from FNA specimens does however not exclude or rule out an aggressive lymphoma or histiocytic disorder. A review of RDD cases in Africa shows the disparity in definitive clinical diagnosis of mycobacterium tuberculosis infection, confirmed in one case, and the number of patients empirically started on anti-tuberculous therapy (n=17). This disparity speaks to the importance of tuberculosis as a differential for lymphadenopathy in patients where there is a high burden of tuberculosis infection. Empiric anti-tuberculous therapy in patients with significant lymphadenopathy may also result in delays of a lymphoma diagnosis as both diseases have overlap in presentations. Rosai-Dorfman-Destombes disease, although a rare disorder, should be considered as a differential diagnosis in patients with massive bilateral cervical lymphadenopathy and is confirmed with accompanying pathological changes on microscopic and immunohistochemical examination of biopsy specimens [1].

## Conclusion

Rosai-Dorfman-Destombes, although a rare disorder, should be considered as a differential diagnosis in patients with massive bilateral cervical lymphadenopathy and is confirmed with accompanying pathological changes on microscopic and immunohistochemical examination of biopsy specimens [1]. The role of infection, particularly HIV infection, is considered to be a possible contributor to the pathogenesis of RDD and HIV testing in patients from areas of high HIV endemicity with co-existing RDD should be undertaken.

### What is known about this topic

- *Rosai-Dorfman-Destombes (RDD) disease, although a rare non-Langerhans cell histiocytosis, presents with massive lymphadenopathy and the diagnosis thereof may pose a diagnostic challenge when a number of other pertinent differential diagnoses are concerned (including mycobacterium tuberculosis and lymphoma);*
- *Although a number of case reports have described the disease presentation, epidemiology and limited treatment options available on the African continent, few have described postulates of particular disease triggers in particular the role of Human immunodeficiency virus (HIV) infection;*
- *Global data from the United Nations Programme on HIV/AIDS (UNAIDS) noted Eastern and Southern Africa as major contributors to the most number of new HIV infections in 2021, with only a few case reports of RDD from African literature with confirmatory testing for the human immunodeficiency virus infection; this short communication highlights the paucity of HIV testing in the setting of massive lymphadenopathy*

### What this study adds

- *This study highlights and brings to light the need for rapid tissue diagnostics in patients with lymphadenopathy, as well as the consideration of diagnoses that are non-infective;*
- *By conducting literature searches of RDD in Africa and presenting two cases of RDD to illustrate the treatment challenges, it will hopefully provide insight and awareness for resource-poor centres in recognizing this disease*.
